# Glaucoma associated with iridocorneal endothelial syndrome in 203 Indian subjects

**DOI:** 10.1371/journal.pone.0171884

**Published:** 2017-03-10

**Authors:** Premanand Chandran, Harsha L. Rao, Anil K. Mandal, Nikhil S. Choudhari, Chandra S. Garudadri, Sirisha Senthil

**Affiliations:** VST Glaucoma Centre, L V Prasad Eye Institute, Hyderabad, Telangana, India; Bascom Palmer Eye Institute, UNITED STATES

## Abstract

**Purpose:**

To report the demographic profile, clinical features, and prevalence of glaucoma and its management in patients with Iridocorneal endothelial (ICE) syndrome.

**Methods:**

Retrospective review of 203 consecutive subjects with ICE syndrome at a tertiary eye care centre between January 1988 and June 2013.

**Results:**

ICE syndrome was present in 223 eyes of 203 subjects, 124 (61%) were female and 79 (39%) were male. The median age at presentation was 43 years (1^st^ (Q_1_) and 3^rd^ (Q_3_) quartile; 34, 51 years). ICE syndrome was unilateral in 183 (90%) subjects, and bilateral in 20 (10%) subjects. The most common clinical variant was progressive iris atrophy (PIA, 115; 52% eyes), followed by Chandler syndrome (CS, 87; 39% eyes) and Cogan-Reese syndrome (CRS, 21; 9% eyes). Glaucoma was found in 156 eyes (70%) at presentation and the median (Q_1_, Q_3_) intraocular pressure in eyes with glaucoma was 24 (16, 38) mm Hg. Seven eyes developed glaucoma during the follow-up period, increasing the percentage of eyes with glaucoma to 73%. Intraocular pressure was managed medically in 81 eyes (50%) and the other 82 eyes (50%) required surgical intervention. Corneal edema was present in 124 eyes (56%) of which, 32 eyes (14%) required keratoplasty.

**Conclusions:**

In our study on ICE syndrome in Indian population, the presentation was predominantly uniocular and more common in middle aged women. Progressive iris atrophy was the most common clinical variant. ICE syndrome was associated with glaucoma in over 70% of the eyes and half of the eyes had corneal edema.

## Introduction

The iridocorneal endothelial (ICE) syndrome is a disease spectrum which includes Chandler syndrome (CS), Progressive iris atrophy (PIA) and Cogan-Reese syndrome (CRS). The clinical variants are distinguished primarily on the basis of the changes in the iris. Progressive or essential iris atrophy is used when there is predominant iris involvement in the form of extensive iris stromal atrophy and hole formation. Predominant corneal involvement with normal or mild iris stromal atrophy are classified as Chandler syndrome, and those with characteristic iris nodules with any degree of iris stromal atrophy are classified as Cogan-Reese syndrome [1]. The term ICE syndrome comprising of all the three clinical variants was coined by Yanoff in 1979 [2].

The ICE syndrome is a progressive disease of the corneal endothelium and is associated with variable features like corneal edema, hammered silver appearance of the posterior corneal surface, iris abnormalities ranging from corectopia to polycoria or iris nodules, broad based peripheral anterior synechiae (PAS) and secondary glaucoma [3]. It typically presents as a unilateral condition, but bilateral or subclinical abnormalities in the corneal endothelium of the fellow eye are not uncommon [4, 5]. It usually manifests in the early to middle adulthood and is seen more frequently in females [3, 6].

The basic pathology lies in the corneal endothelium which proliferates and migrates across the anterior chamber angle and on to the anterior surface of the iris. Contraction of this membrane leads to iris changes, PAS and secondary glaucoma [7]. ICE syndrome is an acquired condition, with unclear etiology, a few reports postulating involvement of Herpes simplex virus in the etiopathogenesis of the disease [8]. The two major vision threatening sequelae of ICE syndrome are glaucoma and corneal decompensation. The severity of the disease and the predominant clinical variants seem to differ among various ethnicities [3, 6, 9].

There are only a few case series published on this condition, largest being Shields et al [6], in 82 eyes from Caucasian population, and Teekhasaenee and Ritch [9], in 60 eyes from southeast Asian population. Currently, there is no literature on ICE syndrome from Indian cohort. The objective of the current study is to report the demographic features of the patients with ICE syndrome in the largest series till date in Indian population. Also to report the prevalence of glaucoma associated with ICE syndrome.

## Materials and methods

We retrospectively analyzed the clinical data of all subjects diagnosed with ICE syndrome at our institute over a period of 25 years, between January 1988 and June 2013. The study was approved by the L V Prasad eye institute’s ethics committee. Patients with Axenfeld-Rieger syndrome, uveitis, ocular trauma and other causes of primary or secondary angle closure were excluded from the study.

ICE syndrome was diagnosed based on the typical characteristic feature of hammered-silver appearance of the posterior corneal surface or corneal edema and iris stromal abnormalities ranging from corectopia to polycoria, with or without iris nodules and broad based PAS. The subjects were classified into subtypes of ICE syndrome primarily on the basis of iris abnormalities. Subjects with normal or mild iris stromal atrophy with predominant corneal involvement were classified as CS, those with predominant iris involvement in the form of extensive iris stromal atrophy and hole formation were classified as PIA and those with characteristic iris nodules with any degree of iris stromal atrophy were classified as CRS.

The data recorded were age, gender, number of anti-glaucoma medications (AGM), prior surgical intervention(s), laterality, visual acuity, corneal and iris changes, intraocular pressure (IOP), gonioscopic findings, optic disc changes, and specular microscopy and visual field data wherever possible.

Glaucoma was diagnosed if the IOP is >21 or ≤21 mm Hg on anti-glaucoma medications/previous glaucoma filtering surgery with glaucomatous optic nerve damage in the form of rim thinning, notching, nerve fiber layer defect or asymmetric disc cupping (difference in cup to disc ratio >0.2 between 2 eyes) in the absence of inter-eye asymmetry in the size of the optic disc. We defined ocular hypertension as eyes with IOP >21 mm Hg with no disc or field changes. In secondary glaucoma’s, raised IOP >21 mm Hg is considered as glaucoma and we rarely use the term secondary ocular hypertension. In the current study, we have combined eyes with glaucoma and OHT, and have referred to it as glaucoma in the entire manuscript.

On gonioscopy the extent of PAS was graded as absent, mild (<90°), moderate (90°to 180°), severe (>180° to 270°) or extensive (>270°). Cornea was graded as clear or edematous based on the corneal clarity and the visibility of the iris texture and the pupilary details. Humphrey visual fields were classified as mild, moderate and severe defects according to the Hoddapp, Parrish, and Anderson classification [10].

Statistical analysis predominantly consisted of descriptive analysis. Descriptive statistics included median (50^th^ percentile) with 25^th^ percentile (1^st^quartile given as Q_1_) and 75^th^percentile (3^rd^ quartile given as Q_3_) values for continuous variables. Categorical variables were summarized as percentages. Non-parametric tests (Kruskal Wallis and Wilcoxon rank sum test) were used to compare continuous variables between the subtypes of ICE syndrome and Chi square test to compare percentages. Statistical analysis was performed using commercial software (Stata ver. 11.2; StataCorp, College Station, Tx).

## Results

During the study period, 223 eyes of 203 subjects were diagnosed with ICE syndrome. The complete data is available as S1 Table. The patient characteristics are shown in Table 1.Median age at presentation was 43 years (Q_1_Q_3_; 34, 51 years); 124 (61%) subjects were female and 79 (39%) were male. Visual acuity at presentation ranged from no perception of light to 20/20. The median presenting visual acuity was worse in patients with Chandler variant compared to PIA (p<0.001, Wilcoxon rank sum test) and CRS (p = 0.03, Wilcoxon rank sum test). The median follow-up period was 18 months (Q_1_Q_3_; 0.1, 57 months). ICE syndrome was unilateral in 183 subjects (90%), of which the right eye was involved in 82 subjects and left eye in 101 subjects. In unilateral cases, PIA was the commonest variant accounting for 96 eyes followed by CS in 67 eyes and CRS in 20 eyes. Bilateral involvement was seen in 20 subjects (10%). Among the eyes with bilateral involvement, six subjects had CS in both the eyes and five subjects had PIA in both the eyes. The remaining nine subjects with bilateral involvement had a combination of two of the three clinical variants in either eye. Seven subjects had CS in one eye and PIA in the other eye, one subject had PIA in one eye and CRS in the other eye, and one subject had PIA in one eye and other eye had specular changes typical of ICE syndrome with no corneal edema or iris involvement (subclinical involvement). Including the unilateral and bilateral subjects, there were 115 eyes with PIA (52%), 87 eyes with CS (39%) and 21 eyes with CRS (9%).

**Table 1:** Characteristics of patients with ICE syndrome.

Characteristic	Entire patient population	PIA (115 eyes)	CS (87 eyes)	CRS (21 eyes)	P*
Age (Median, Q_1_Q_3_)	43 (34, 51)	42 (33, 50)	45 (35, 54)	48 (36, 50)	0.06
VA (Median, Q_1_Q_3_)	20/80 (20/30, CF 1m)	20/40 (20/20, 20/400)	CF 1m (20/60, CF 1m)	20/80 (20/20, 20/400)	0.001
IOP (Median, Q_1_Q_3_)	18 (14, 32)	20 (14, 38)	18 (15, 34)	26.5 (18, 35)	0.007

PIA, indicates progressive iris atrophy; CS, Chandler syndrome; CRS, Cogan Reese syndrome; VA, visual acuity; Q_1,_ 1^st^quartile; Q_3,_ 3^rd^ quartile; IOP, intraocular pressure; CF 1m, counting fingers at 1 meter.

* p value derived from Kruskal Wallis test.

The median presenting IOP in 210 eyes was 18 mm Hg (Q_1_Q_3_; 14, 32 mm Hg) and 120 of these eyes were on AGM. Intraocular pressure could not be measured in 13 eyes (6%) due to severe corneal edema. The median presenting IOP irrespective of anti-glaucoma treatment was highest in patients with CRS compared to PIA (p = 0.04, Wilcoxon rank sum test) and CS (p = 0.006, Wilcoxon rank sum test).

### Corneal findings

Clear cornea was present in 99 eyes (44%) and corneal edema was seen in 124 eyes (56%), of these 28 eyes had severe corneal edema needing keratoplasty. The corneal edema was significantly more common in eyes with CS (82; 66% eyes), compared to eyes with PIA (34; 28% eyes) and CRS (8; 6% eyes, *P*<0.001, Chi square test). The level of IOP did not differ between eyes with or without corneal edema (*P* = 0.57, Wilcoxon rank sum test). In unilateral cases, corneal edema was more often seen in females (63; 62%) than in males (39; 38%). On the contrary, in bilateral cases, corneal edema was more often seen in males (15; 68%) than in females (7; 32%). However, both these gender differences did not reach statistical significance (*P* = 0.4 and 0.27; respectively, Chi square test).Among the eyes with clear cornea, the specular microscopy data on endothelial cell density was available in 39 eyes. The median endothelial cell density in these eyes was 2112 cells/mm^2^ (Range; 858, 3413 cells/mm^2^).

During the follow-up period, 4 more eyes (in addition to the 28 at presentation) had worsening of corneal edema and all these 32 eyes (14%) required keratoplasty. Among these were23 eyes (26%) with CS, 7 eyes (6%) with PIA and 2 eyes (9%) with CRS that underwent keratoplasty. Of these, 6 eyes underwent penetrating keratoplasty (PK), 11 eyes underwent combined PK with cataract surgery, 6 eyes underwent Descemet stripping endothelial keratoplasty (DSEK) and 9 eyes required combined DSEK with cataract surgery.

### Iris findings

Corectopia was present in 210 eyes (94%) (115 with PIA, 74 with CS and 21 with CRS). The pupils were deviated towards the site of PAS. Polycoria was present in 129 eyes (58%) (115 with PIA and 14 with CRS). The iris nodules in CRS were round, irregular and hyper pigmented lesions. No iris change was noted in 13 eyes (15%) with CS.

### Anterior chamber angle findings

In 143 eyes gonioscopy was possible and in the remaining 80 eyes, the angle details were obscured by corneal edema. Of the 143 eyes with gonioscopic documentation, 5 eyes had open angles with no PAS, 8 eyes had mild PAS, 20 eyes had moderate PAS, 33 eyes had severe PAS and 77 eyes had extensive PAS. The synechiae were generally broad and extended anterior to the Schwalbe’s line and were visible on routine slit lamp examination. Peripheral anterior synechiae more than 180° in extent occurred in 64 eyes with PIA (56%), 32 eyes with CS (37%) and 14 eyes with CRS (67%). The extent of synechial angle closure did not vary significantly with the subtype of ICE syndrome (*P* = 0.2; Chi square test).

### Glaucoma in ICE syndrome

Of 223 eyes with ICE syndrome, 156 eyes (70%) had glaucoma at presentation. Increased IOP without disc damage (Ocular hypertension) was noted in 74 eyes and 82 eyes had glaucomatous disc damage. During the follow up period, 7 more eyes developed glaucoma, increasing the total number to 163 eyes (73%) with glaucoma.

The Median (Q_1_Q_3_) IOP at presentation among the eyes with glaucoma secondary to ICE syndrome was 24 (16, 38) mm Hg. About two third eyes with glaucoma (120 eyes; 77%) were on AGMs at presentation, the median number of AGM was 2 (Q_1_Q_3_: 1, 2.5). At presentation, 34 eyes had undergone prior glaucoma filtering surgery. Automated perimetry was performed in 42 eyes with sufficient visual acuity and corneal clarity. The median mean deviation (MD) in these 42 eyes was -10.5 dB (Q_1_Q_3_; -5.3, -24 dB). Thirteen eyes had mild visual field loss, 8 eyes had moderate loss, and 21 eyes had severe loss.

Intraocular pressure was managed medically in 81 eyes (50%) and the other 82 eyes (50%) required surgical intervention. Surgical intervention was required for the control of IOP in 54% of subjects with PIA, 47% of CR and 45% of CS, and the others were managed medically. At the last follow-up, 82 eyes underwent 114 surgical interventions (87 trabeculectomy, 7 Ahmed glaucoma valve (AGV) implant and 20 Trans scleral cyclophotocoagulation) for the control of IOP. Of the 87 filtering procedures, 54 eyes had trabeculectomy and 33 eyes had mitomycin C (MMC) augmented trabeculectomy. Of the 82 eyes that underwent surgical intervention, 22 eyes (27%) required a second, 6 eyes (7%) a third, 3 eyes (4%) a fourth and 1 eye (1%) required a fifth surgical procedure to control the IOP.

**Fig 1:**
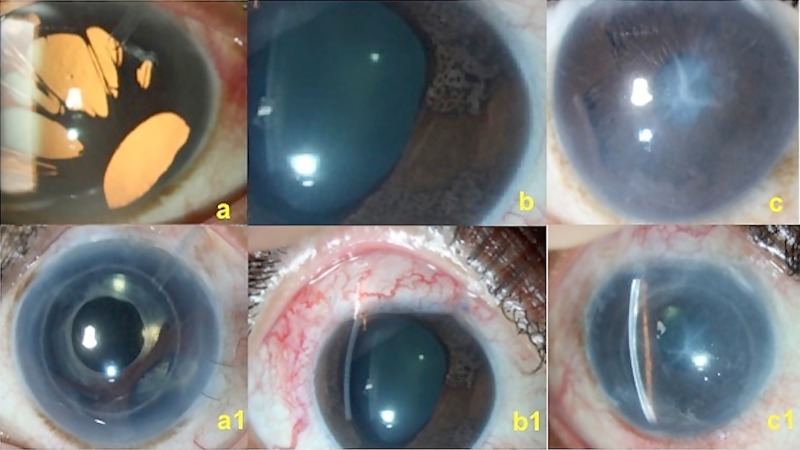
Clinical photograph showing the 3 variants of ICE syndrome. (a) Eye with progressive iris atrophy after AGV implantation, (a1) shows the same eye after DSEK and intraocular lens implantation. (b) Eye with Cogan-Reese syndrome, (b1) same eye after trabeculectomy with MMC. (c) Eye with Chandler syndrome, (c1) same eye after trabeculectomy with MMC and DSEK.

The comparison of the clinical features of the 3 subtypes of ICE syndrome is shown in Table 2 and Fig 1 shows the 3 clinical variants of ICE syndrome.

**Table 2:** Comparison of clinical features of the 3 subtypes of ICE syndrome.

	PIA	CS	CRS	P
	(115 eyes)	(87 eyes)	(21 eyes)	
**Corneal edema**				
No. (%) of eyes	34 (29)	82 (93)	8 (40)	<0.001
**Iris changes**				
(No. (%) of eyes)				
Corectopia	115 (100)	74(85)	21 (100)	<0.001
Polycoria	115 (100)	0	14 (67)	
Iris nodules	0	0	21 (100)	
**Glaucoma**				
No. (%) of eyes	88 (76)	58 (67)	17 (81)	0.21

PIA, indicates progressive iris atrophy; CS, Chandler syndrome; CRS, Cogan Reese syndrome.

## Discussion

In ICE syndrome, abnormal corneal endothelial cells proliferate and grow over the anterior chamber angle and iris. The three clinical variants vary in their involvement of the cornea, iris changes and glaucoma. In our cohort of ICE syndrome, the condition was predominantly unilateral, women were more commonly affected and progressive iris atrophy was the most common clinical variant. The most common complication noted in eyes with ICE syndrome was secondary glaucoma followed by corneal edema.

ICE syndrome commonly occurs in young adults, with increased prevalence in women, the median age at presentation in our series was 43 years, which was similar to the reports from Wilson et al (41.5 years) [3], Teekhasaenee et al (43.7 years) [9], and Shields et al (38.6 years) [6].Majority of the subjects with ICE syndrome were women (61%), which was similar to other published series, Wilson et al (62.2%) [3], Teekhasaenee et al (66.7%) [9], and Shields et al (68.3%) [6].

ICE syndrome was unilateral in 90% of our subjects. We also noted bilateral involvement in 10% of the subjects, of which 8 subjects had two different clinical variants in either eye. Hemady et al [4], and Huna et al [5], have also reported bilateral involvement in ICE syndrome, among which Huna et al [5], have reported two different clinical variants in the same subject. Presentation of two different variants in two eyes could be due to the fact that ICE syndrome is a spectrum and there can be an overlap in the clinical presentation between the two eyes. Teekhasaenee et al [9], in their series of 60 subjects, have reported subclinical involvement in the contralateral eye of 14 subjects. We could identify only one eye with subclinical ICE syndrome and it is possible that we have missed at least a few due to a non uniform availability of the endothelial data in the contra lateral eye of the patients with unilateral ICE syndrome.

Progressive iris atrophy (52%) was the most common presentation in our series, as was also shown by Laganowski et al [11].However, the Chandler variant was the predominant type as reported by Wilson et al in Caucasian population [3], and Cogan-Reese variant was the commonest in a report by Teekhasaenee et al among Southeast Asian population [9]. This suggests possible ethnic differences in the presentation of the disease.

As expected, subjects with Chandler syndrome had worse visual acuity than CRS and PIA. About one third eyes in our series presented with corneal edema despite normal IOP and a majority of these eyes had CS. This could be explained by the increased prevalence of corneal endothelial dysfunction in CS unrelated to high IOP. These findings were similar to the series reported by Wilson et al [3]. The iris changes were confined to corectopia in majority of the eyes with CS. On the other hand, the iris changes were more extensive in other two subtypes of ICE syndrome.

Iris changes in the form of polycoria were more common in our population compared to the other two populations (P<0.05, pair wise comparisons by t-test) and corectopia was more common in our population compared to the Caucasian population (P<0.05, t-test). This is possibly due to the higher prevalence of progressive iris atrophy in our population. Comparison of demographic profile and clinical features of ICE syndrome across various studies are given in Table 3.

**Table 3:** Comparison of ICE syndrome between present series and other series in Southeast Asian and Caucasian patients.

Parameter	Present series (Indian)	Teekhasaenee and Ritch (Southeast Asian)	Shields et al (Caucasian)
**Total No. eyes (No. of patients)**	223 (203)	60 (60)	82 (82)
**Age at onset/at presentation in years**	43 (34, 51)^a^	43.7 ± 12.3^b^	38.6 (6–58)^c^
**Percent female**	61	66.7	68.3
**Variant**	PIA (52.4%) > CS > CRS^d^	CRS (63.3%) > CS > PIA	CS, PIA > CRS^e^
**Corneal edema: No. (%) of eyes**	124 (55.7%)^d^	25 (41.7%)	41 (50)
**Iris changes: No. (%) of eyes**			
Corectopia	210 (94.1%)	59 (98.3%)	58 (70.7%)
Polycoria	129 (57.8%)	13 (21.6%)	20 (24.3%)
Ectropion uvea	NA	42 (70%)	23 (28%)
**Glaucoma: No. (%) of eyes**	156 (69.9%)	46 (76.7%)	63 (76.8%)

PIA, indicates progressive iris atrophy; CS, Chandler syndrome; CRS, Cogan Reese syndrome;

^a^, median (1^st^ and 3^rd^ quartile);

^b^, mean ± standard deviation;

^c^, mean (range);

^d^, in unilateral cases;

^e^, eyes were not divided into CS and PIA.

The prevalence of glaucoma in ICE syndrome was reported to range from 46% to 82% [6, 11, 12]. In our series the prevalence of glaucoma was 73%. Due to its refractive nature, medical management is considered ineffective in subjects with ICE syndrome [11, 13–15]. In our series, IOP was controlled with medical management in half the eyes with glaucoma (50%) which was higher than other series which ranged from 12–40% [6, 9, 11].The probable reasons for the variable response could be due to differences in the disease severity across studies and also possibly due to better IOP lowering medications available over the past 2 decades. Surgical intervention for IOP control was required in 50% of the eyes and majority had trabeculectomy with or without antimetabolite use. As was reported earlier, the success of primary trabeculectomy with MMC in our series was 55% at 5 years [16]. Close to a quarter (27%) of the eyes that underwent trabeculectomy needed second surgery for IOP control and refractory cases underwent Ahmed glaucoma valve implantation.

Glaucoma was probably more refractory in PIA than CRS and CS in our series, the number of eyes needing surgical intervention for glaucoma were more in eyes with progressive iris atrophy compared to the other two variants (although not statistically significant), which possibly suggests refractory and progressive nature of this disease variant. Glaucoma was reported to be less severe and easier to manage in CS by several investigators as the pathology in this variant predominantly involves the cornea [1, 3, 17], however this was not so in another study [11]. In the current study, glaucoma in Chandler variant was less severe than PIA, however, the difference was not statistically significant.

Our study has several limitations besides the known limitations of any retrospective study. Corneal endothelial details were not available in a significant proportion of eyes which did present with clear cornea or in the contralateral eye of the patients with unilateral ICE syndrome. The grading of corneal edema was clinical and subjective. However, our study is the largest series till date on this disorder reporting on the spectrum of disease in different population. Also, both eyes of a few patients were included in our study and we did not account for this in our analysis. However, the number of patients whose both eyes were analysed was small (20 of 203 patients) and we believe that not accounting for the two eye inclusion of this small number of patients is unlikely to have affected the results.

## Conclusions

In our cohort, ICE syndrome was unilateral in majority of the subjects, middle aged women were more commonly affected, and progressive iris atrophy was the most common variant. The two major sight threatening complications were secondary glaucoma which was present in 73% of the eyes and corneal edema in 56% of the eyes. The glaucoma was refractory and needed surgical intervention in 50% of the eyes and 14% of the eyes with corneal edema needed corneal transplant.

## Supporting information

S1 Table Complete data is available as supporting information Table 1. (XLSX)

## References

[1] Shields MB. Progressive essential iris atrophy, Chandler's syndrome, and the iris nevus (Cogan-Reese) syndrome: a spectrum of disease. Surv Ophthalmol 1979;24:3–20. 483159 10.1016/0039-6257(79)90143-7

[2] Yanoff M. Iridocorneal endothelial syndrome: unification of a disease spectrum.Surv Ophthalmol 1979;24:1–2. 483158 10.1016/0039-6257(79)90142-5

[3] Wilson MC, Shields MB. A comparison of the clinical variations of the iridocorneal endothelial syndrome. Arch Ophthalmol 1989;107:1465–8. 2803093 10.1001/archopht.1989.01070020539035

[4] Hemady RK, Patel A, Blum S, Nirankari VS. Bilateral iridocorneal endothelial syndrome: case report and review of the literature. Cornea 1994;13:368–72. 7924340 10.1097/00003226-199407000-00015

[5] Huna R, Barak A, Melamed S. Bilateral iridocorneal endothelial syndrome presented as Cogan-Reese and Chandler's syndrome. J Glaucoma 1996;5:60–2. 8795735

[6] Shields MB, Campbell DG, Simmons RJ. The essential iris atrophies. Am J Ophthalmol 1978;85:749–59. 677202 10.1016/s0002-9394(14)78101-2

[7] Campbell DG, Shields MB, Smith TR. The corneal endothelium and the spectrum of essential iris atrophy. Am J Ophthalmol 1978;86:317–24. 717494 10.1016/0002-9394(78)90232-5

[8] Alvarado JA, Underwood JL, Green WR, Wu S, Murphy CG, Hwang DG et al Detection of herpes simplex viral DNA in the iridocorneal endothelial syndrome. Arch Ophthalmol 1994;112:1601–9. 7993217 10.1001/archopht.1994.01090240107034

[9] Teekhasaenee C, Ritch R. Iridocorneal endothelial syndrome in Thai patients: clinical variations. *Arch Ophthalmol* 2000;118:187–92. 10676783 10.1001/archopht.118.2.187

[10] Hodapp E, Parrish RK II, Anderson DR. Clinical decisions in glaucoma. St. Louis: The CV Mosby Co;1993:52–61.

[11] Laganowski HC, Kerr Muir MG, Hitchings RA. Glaucoma and the iridocorneal endothelial syndrome. Arch Ophthalmol 1992;110:346–50. 1543451 10.1001/archopht.1992.01080150044025

[12] Hirst LW, Quigley HA, Stark WJ, Shields NB. Specular microscopy of irido-corneal endothelial syndrome. Aust J Ophthalmol 1980;8:139–46. 7447799 10.1111/j.1442-9071.1980.tb01672.x

[13] Rodrigues MM, Streeten BW, Spaeth GL. Chandler's syndrome as a variant of essential iris atrophy. A clinicopathologic study. Arch Ophthalmol 1978;96:643–52. 646692 10.1001/archopht.1978.03910050339009

[14] Hetherington J Jr. The spectrum of Chandler's syndrome. Ophthalmology 1978;85:240–4. 307212 10.1016/s0161-6420(78)35678-5

[15] Kidd M, Hetherington J, Magee S. Surgical results in iridocorneal endothelial syndrome. Arch Ophthalmol 1988;106:199–201. 3341974 10.1001/archopht.1988.01060130209027

[16] Chandran P, Rao HL, Mandal AK, Choudhari NS, Garudadri CS, Senthil S. Outcomes of primary trabeculectomy with mitomycin-C in glaucoma secondary to iridocorneal endothelial syndrome. J Glaucoma 2016;25:e652–6. doi: 10.1097/IJG.0000000000000268 25943731 10.1097/IJG.0000000000000268

[17] Richardson TM. Corneal decompensation in Chandler's syndrome. A scanning and transmission electron microscopic study. Arch Ophthalmol 1979;97:2112–9. 508178 10.1001/archopht.1979.01020020430003

